# Five actions for five people: emergency cesarean section protocol

**DOI:** 10.1186/s12884-023-05591-9

**Published:** 2023-04-20

**Authors:** Paolo Mannella, Federica Pancetti, Andrea Giannini, Eleonora Russo, Magdalena Montt-Guevara, Tommaso Simoncini

**Affiliations:** Department of Clinical and Experimental Medicine, University of Pisa, Via Roma 67, Pisa, 56126 Italy

## Abstract

**Objective:**

The scope of this work is to evaluate an operative protocol for emergency C-section to improve teamwork and reduce surgical setup time.

**Methods:**

Sixty-six health care operators working together in the delivery ward (gynecologists, midwives, anesthesiologists) simulated an emergency scenario applying a “five actions for each operator” protocol. For each simulation, the decision to delivery interval was considered and the perception of each operator as a team worker was analyzed with specific tests.

**Results:**

The “five actions for five people” protocol significantly reduces the decision to delivery interval (p < 0.001) for emergency C-section. At the same time, a simple and codified scheme improves communication among team members, avoids overlapping roles. Indeed, all the operators become more aware of being helpful to the team (p < 0.001).

**Conclusion:**

The use of a standardized, simple, and immediately usable protocol improves the performance of the delivery room team in terms of the urgency and quality of the operator’s participation in the event. Procedures of this type should be favored within emergency obstetric settings.

**Trial registration number:**

CEAVNO 19-01-23. Local ethical Committee (COMITATO ETICO REGIONALE PER LA SPERIMENTAZIONE CLINICA - Sezione autonoma Area Vasta Nord Ovest -CEAVNO) approved this study as simulation training study. All the operators participated voluntary during their working time.

## Introduction

Performing an emergency cesarean section requires the activation of strict operative protocols. However, this is often not sufficient [1]. Delays can occur due to simple communication failures despite adherence to protocols. [2]. Sometimes, conflicts arise in the actions of individual actors, especially in emergency situations. [3]. These actions are counterproductive because they hinder the work of others, even if they are necessary but not at that moment [4]. Significant loss of time is the final result.

The concept of time in obstetrics is essential, and often the definition of urgency, the 30-minute rule, is not satisfactory [5]. There are clinical conditions, fortunately of low incidence, such as abruptio placentae, umbilical cord prolapse, and uterine rupture, in which a cesarean section must be performed quickly because of ‘’immediate threat to the life of the woman or fetus’’ [6–8]. In these cases, the time taken from the decision to perform a cesarean section to the skin incision must be much shorter [9–13].

Therefore, it is critical to develop a series of quick, precise, and coordinated actions among team members. Such actions follow a temporal order, not according to their importance, but according to the necessary sequence that matches those of the team. The team leader must coordinate the entire team like a conductor of an orchestra, and musicians have a perfect understanding of timing and the order of their own actions.

Each childbirth hospital should adopt established protocols for emergency cesarean section. It is clear that training can contribute to improve maternal and perinatal outcomes, compared with no training [14]. However, there are no recognized protocols that define the necessary actions for each operator and for each event.

The purpose of this work is to establish a sequence of actions to be performed by each operator in an emergency situation which requires emergency cesarean section. Once the various sequences were agreed, we then carried out a series of simulations to verify the time taken from the moment of the decision to the time of incision of the patient’s skin. We then evaluated how each operator responded to this protocol and how it fit with the teamwork concept.

## Materials and methods

Sixty-six health operators working in the maternity ward of the University of Pisa participated in this study. The whole project took about 8 months for several reasons that we will explain later. The workers were divided according to their role: gynecologists (12), midwives (30), anesthesiologists (12), and healthcare assistants (12). In our institute, midwives work in the obstetrical surgical room as operating room nurses and scrub nurses.

Before the simulations, each operator was given a test in which they could freely and anonymously answer 5 questions with a value from 1 (minimum) to 10 (maximum) (Table 1).

**Table 1:** Self-assessment filled out by each operator. 1 is considered minimum (fair), 10 maximum (optimal)

How effective do you think the communication between operators is?									
1	2	3	4	5	6	7	8	9	10
Regarding the teamwork, how useful executing specific task is?									
1	2	3	4	5	6	7	8	9	10
How much do you think you have negatively interfered with the work of other operators? (reminders, solicitations, overlapping, physical impediment)									
1	2	3	4	5	6	7	8	9	10
How much do you think you have helped the team?									
1	2	3	4	5	6	7	8	9	10
How useful do you think simulating is?									
1	2	3	4	5	6	7	8	9	10

In the case of the midwives, they filled out the forms according to the role they have, operating room nurse or scrub nurse.

The scenario selected for simulation was a massive placental abruption in a full-term pregnant woman who presented to the emergency room with heavy bleeding. All the sessions were conducted during the normal rotation and without the knowledge of the staff. We routinely use the simulation to re-train our operators. In fact, when the operators saw the mannequin, they did not automatically know what type of simulation was taking place.

In order to avoid repetition of the scenario by the same people who had already done several sessions on this event, it was decided to start the simulation test if at least 4 out of 5 people in the team had never done simulation activities on that particular scenario. This reason justified the time needed to complete the entire project.

Those simulations, in which the midwife was previously played a different role (operating room nurse or scrub nurse), were considered to be valid.

### Scenario design

A pregnant woman at term arrives with massive genitals bleeding at the emergency room with her partner. The midwife notices the urgency and calls the on-call gynecologist who stays in the delivery ward. At the beginning, no one knows that the urgency is a simulation.

The scenario is presented with a bleeding simulator (at least 500 ml) and a cardiotocographic tracing describing fetal bradycardia (about 90 bpm) for more than 10 min with reduced variability (< 5). The SimMom birthing simulator (Laerdal, Gatesville, TX, USA) was also used for this type of simulation; in fact, it was actually used more for its confusion effect than for its technical features.

The on-call gynecologist, after assessing the extent of bleeding and fetal bradycardia, decides to perform an emergency cesarean section.

Time counting starts at the time the on-call physician decides to perform an emergency cesarean section. We only considered the time from decision to incision.

### Experimental design (Fig. 1)

**Fig. 1:**
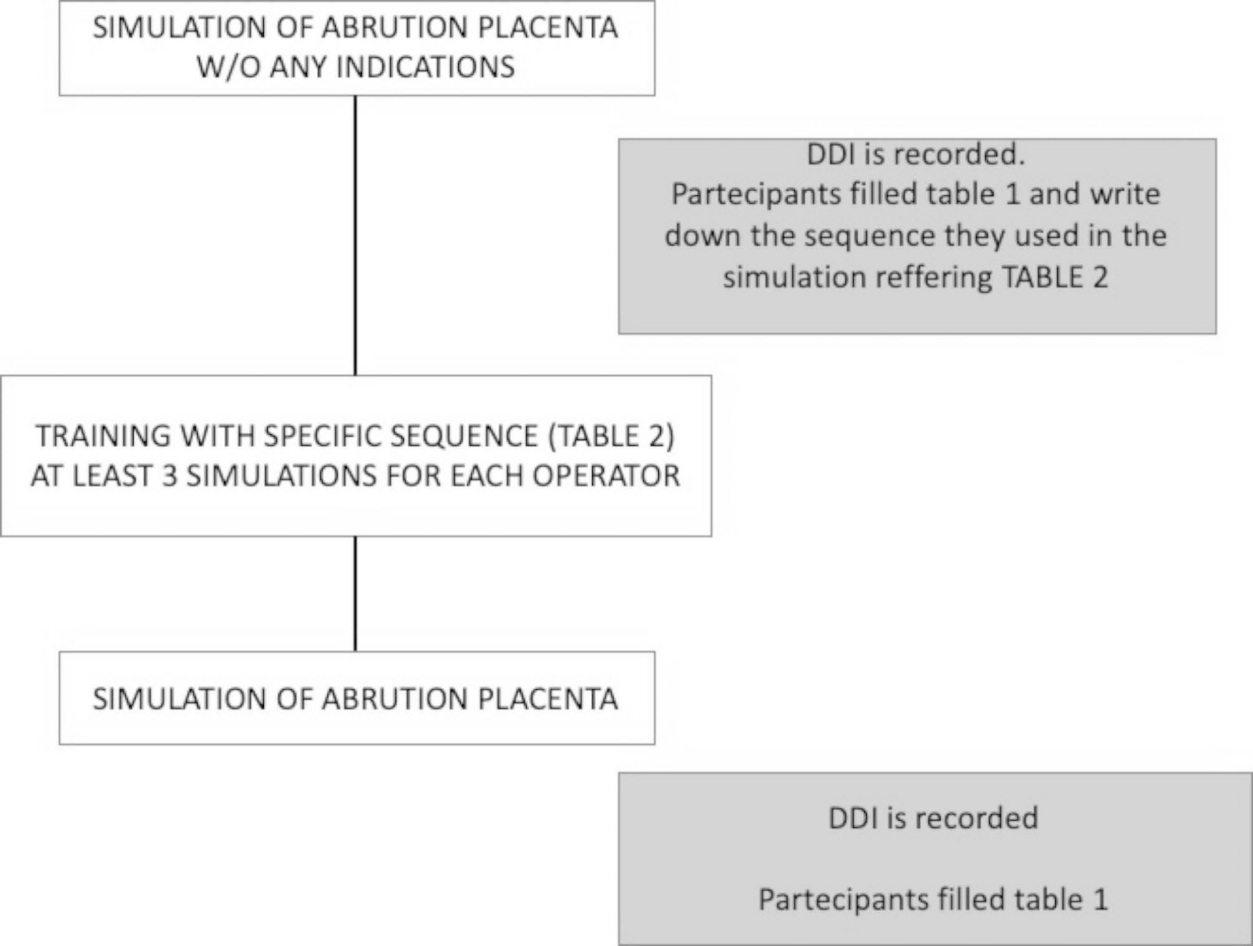
Experimental design

Before the training began, different teams ran a single simulation to find out how long it would take them to complete the scenario, known as the Decision-to-Delivery Interval (DDI). We then asked them to write down the sequence they used in that scenario. After that, we gave them an exact sequence of actions to performed according to their roles, and they were trained (Table 2). Each operator only has to perform five actions. Nothing more.

**Table 2:** 5 actions for each operator which they have to perform sequentially

**Gynaecologist/ Surgeon**	
1	Making decision for eCS
2	Communicating with patient and relatives about eCS and obtaining informed consent
3	Calling for other operators (anaesthesiologists, neonatologists, another midwife, eventual second operator)
4	Preparing for the surgical procedure (wearing surgical mask, overshoes and disposable cap, surgical hand washing, wearing surgical clothes)
5	Surgical site disinfection, bladder catheter application, disinfection and eCS execution.
**Anaesthesiologists**	
1	Going to patient and evaluating which type of anaesthesia perform to
2	Checking allergies and last meal
3	Preparing two vein accesses (16G or 14G)
4	Preparing drug and material for anaesthesia
5	Inducing local or general anaesthesia
**First Midwife (operating theatre nurse)**	
1	Calling second midwife
2	Preparing for the surgical procedure (wearing surgical mask, overshoes and disposable cap, surgical hand washing, wearing surgical clothes)
3	Setting the operating table
4	Dressing the gynaecologist
5	Helping the gynaecologist on eCS
**Second Midwife (scrub nurse)**	
1	Helping first operating room nurse on setting up operating table
2	Connect reservoir for bladder catheter.
3	Helping anaesthesiologist for anaesthesia procedure
4	Completing preparation of operative room (electrosurgical unit, vacuum, drugs etc.)
5	Take neonate to neonatologist
**Healthcare Assistant**	
1	Undressing patient and preparation of the patient for the surgical room
2	Moving patient on the stretcher to the surgical room
3	Positioning of the surgical table, aiding the anaesthesiologist
4	Orientation of the surgical room light
5	Setting of bracelets for mom-neonate identification

The 5 actions were simulated at least 3 times for each operator. When all the operators were aware of the exact pattern to be performed, we again ran a simulation scenario in the same methodologies as expressed before, and again the time DDI was considered.

It is worth noting that although the simulations were performed in teams, each operator had his or her own sequence of actions to perform. This is necessary because an operator could be working in different team due to shift work.

### Statistical analysis

Statistical analysis was performed using GraphPad Prism 7 (GraphPad Software Inc., California, USA). Descriptive statistics were presented as frequencies and percentages for categorical variables, and as means and standard deviations for continuous variables. Data were tested for normal distribution using the D’Agostino & Pearson normality test. In the case of normal distribution, significance was determined via paired t-test, and in the case of non-normal distribution via Wilcoxon matched-pairs signed rank test. A p-value of < 0.05 was considered statistically significant.

## Results

**A detailed sequence of actions improves teamwork.**

Comparing the perception of the work of individual workers, we notice a significant difference when the action is coordinated by an operational scheme rather than carried out independently.

**Fig. 2:**
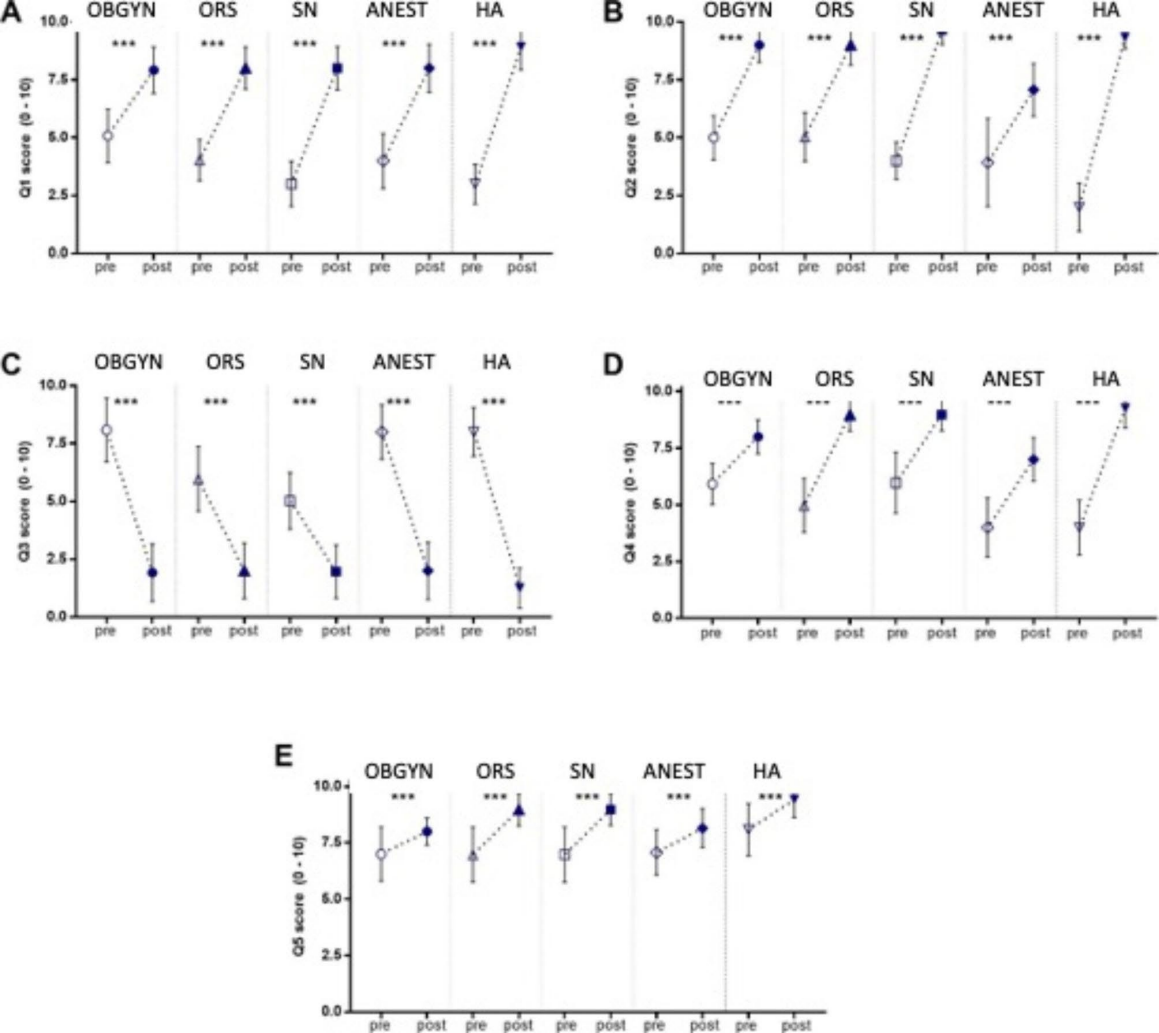
(**A-E).** Impact of simulation on self-assessment test among the different actors in child delivery. The assessment of 5 questions (Q1-Q5) with answers ranging from 0 to a maximum of 10 for the pre and post-test

The creation of simple, codified schemes improves communication between team members; in addition, the use of a specific protocol is considered very useful by participants because it avoids overlapping actions and improves the feeling of having helped the team (all p < 0.001) (Fig. 2A-D). Still, the simulation of these particular scenarios is perceived as very useful to improve one’s own skills p < 0.001 (Fig. 2E).

It should be noted that this significance in the responses applies to all categories of participants (gynecologists, anesthesiologists, midwives, and healthcare assistants).

**The establishment of an operative scheme significantly reduces the decision to delivery interval for emergency cesarean section.**

**Fig. 3:**
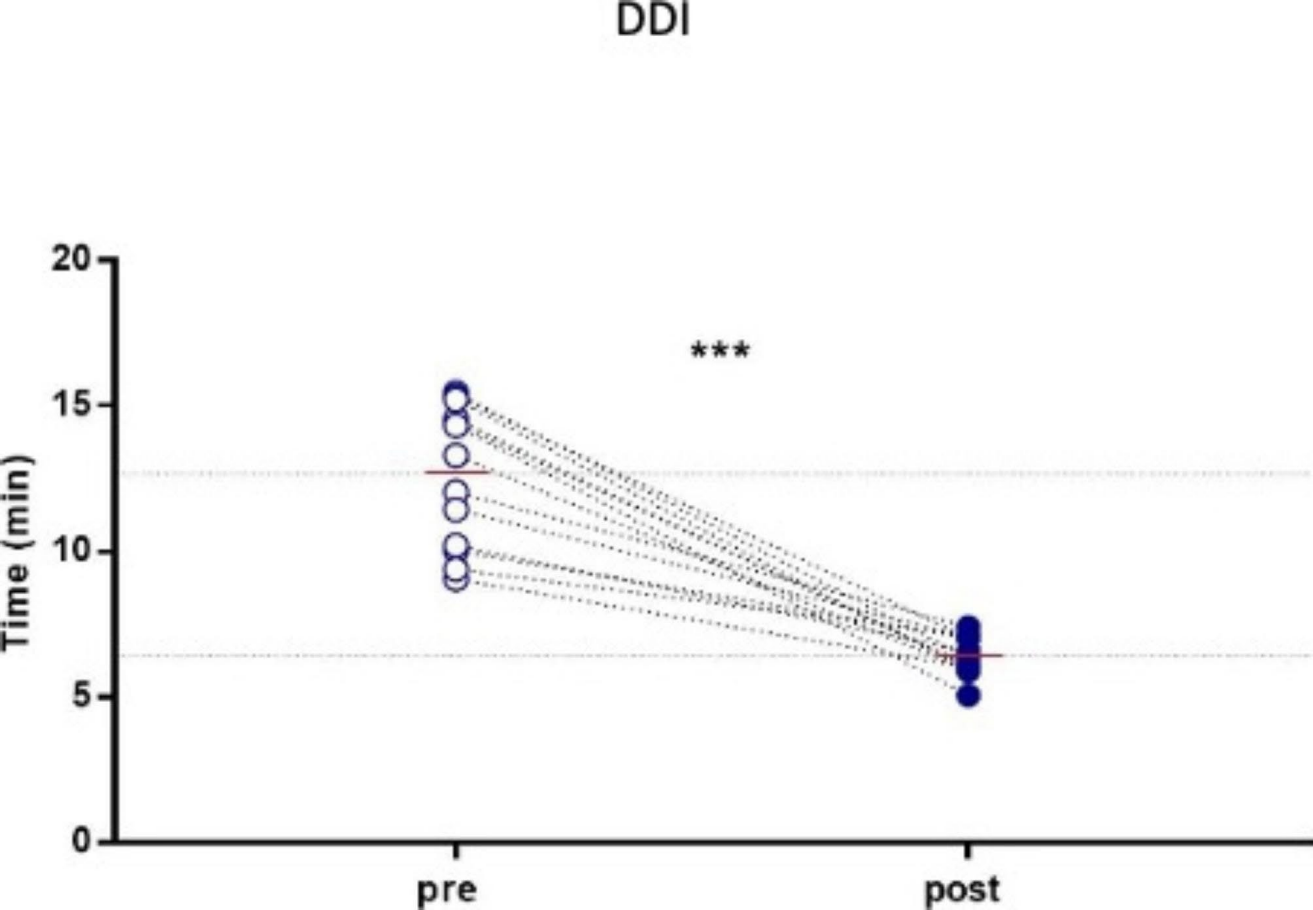
Decision to delivery interval. Data are presented as media ± SD. To identify the differences between pre- and post-simulation assessment, paired t-test or Wilcoxon-matched-pairs signed rank one-tail test was performed. (***: p < 0.001). *OB-GYN = Obstetrician-Gynecologist; ORN = Operative Room Nurse; SN = Scrub Nurse; ANE = Anesthesiologist; HA = Healthcare Assistant*

The main purpose of this program was to reduce the DDI. When operators know exactly what to do and how to do it, they act better individually and as a team. In fact, after at least three simulations for each operator, performed following a well-defined sequence of actions, the result was a significant reduction in the time of the DDI (Fig. 3).

## Discussion

From a planning standpoint, performing an emergency cesarean is complex, and the abilities of the team to work together is maximally stressed.

Good crew resource management is critical to achieving the goals, because even the best surgeons will fail in these circumstances without a close-knit team.

In our study, we used simulation to evaluate each operator’s abilities to rapidly activate the emergency cesarean section procedure for a massive abruption placenta.

The goal of our study was to measure the activity of the team working without considering the own technical skills of each operator.

The use of a sequential protocol, which requires each operator to perform only 5 actions, statistically significantly improves the overall performance of the entire team, as well as the awareness of each participant’ work. After the training, operators have the perception of better communication, of working more harmoniously with the remaining members of the team, avoiding overlapping, calls, and stress, and, in general, they feel much more useful in their actions to achieve the result. The evaluation of the situation awareness of the scenario by the participants with specific and suitable tests is beyond the intentions of this study [15]. However, our data confirm that specific team coordination and cooperation protocols increase the satisfaction of all operators as indicated by the literature [16, 17].

Finally, but certainly, one of the most important results, the time interval between the decision to perform an emergency cesarean section and its execution is significantly reduced.

We purposely chose placental abruption because neonatal outcomes are directly related to the procedure completion time. Indeed, we focused on teamwork rather than specific technical skills. The diagnosis of a massive abruption placenta is easy and the decision to perform an emergency cesarean section was not questionable by operators. Technical skills component is certainly important but, in this study we focused on the rapid setting up of an operating room and preparing the patient for surgery. For this reason, the overall time of the cesarean section procedure (from the incision to skin closure) was not calculated, but only the time to set up the operating field and arrive at the incision (decision to delivery interval).

The time to perform an emergency cesarean section and to have low adverse outcomes is much debated. The best maternal-fetal outcomes depend on the so-called “30-minute rule” [18]. This is the time that must pass between the appearance of the sign (rather massive bleeding etc.) and the delivery of the fetus and includes all the aspects of the C-section, such as the immediate preparation of the room, the arrival of the operators, and up to the extraction of the fetus. The 30-minute interval has been supported by a consensus of expert consensus and is not directly supported by clinical trials or experimental evidence. According to the Guidelines for Perinatal Care published jointly by the American Academy of Pediatrics and ACOG, “Hospitals should have the capability of beginning a cesarean delivery within 30 min of the decision to operate [19]. Many studies have followed the optimal timing, often with conflicting results [9, 11, 20]. Surprisingly, a first evaluation seems to indicate the time factor is not so important or even pejorative [9]. In reality, as the same authors concluded, “even if shorter intervals were associated with low cord pH and higher neonatal risk, however shorter intervals are likely to occur in higher risk cases”[21].

It is not the purpose of this study to discuss the appropriate timing of the intervention, and the type of surgical procedure rather than the more or less stringent indications for the execution of the urgent cesarean section. However, it seems obvious that the less time it takes to set up the operating room and prepare the patient, the more time the operator has to proceed with the extraction of the fetus, especially in the most complicated cases.

On the other hand, the purpose of this protocol is to guarantee rapid times for setting up the operating room to the moment of the incision, beyond the specific indication.

The concept of emergency in obstetrics is also much discussed and “relative”. Therefore, the choice of emergency cesarean section for massive placental abruption is not casual. Placental abruption associated with massive maternal hemorrhage represents one of the main indications for an emergency cesarean section, which is hardly discussed during the procedure and even less on a scientific level.

We demonstrated that a well-coded sequence of actions performed by the staff (gynecologist, anesthesiologist, first midwife, second midwife, healthcare assistant) determines a better synchronization of operators without overlapping tasks that can be unintentionally performed to support the team, but which have an opposite and confounding effect on the final result. Based on this study, each operator is trained to perform only and exclusively 5 actions. These actions have been codified and synchronized as an orchestra conductor synchronizes and directs the input of various instruments during a concert. The adequate preparation of the personnel, train to perform only 5 actions for each role, allows a significant reduction in the time to incision.

Again, teamwork training with simulation improves provider self-satisfaction and patient outcomes. This study confirms and underlines how important teamwork is, in this type of scenario and supports the need to develop teamwork in obstetrics[14]. Human relations, interaction between professional figures and the communicative aspects have long been the subject of study to improve the internal climate but above all to improve team performance [4]. These studies show how important it is the human factor [22]. It is pivotal to recognize the human factor therefore not only as a weakness factor, i.e. at the basis of the error [23, 24] but as an element on which to focus in order to enhance the team’s results [25] .

However, this study presents some points of weakness. First, there is a complete lack of clinical data to report the effects of this simulation program on the care activity. This criticism is very difficult to resolve beyond the perception of the operators themselves and this issue has been largely debated in the literature. Fortunately, performing an emergency cesarean section is not very frequent and the validation of this protocol with clinical data would perhaps take years. At the same time, the interest of the study is to evaluate the effectiveness of a procedure for setting up an operating room, not for the performing a cesarean section, an element that is decidedly operator dependent and that we treated in different programs. Hi-fidelity simulation has been demonstrated to improve competencies [26], define professional competence [27], improve patient outcomes [28], and develop and enhance teamwork[29] [14, 30]. Especially in obstetrics, simulation is increasingly used for training of adverse and low-frequent events, such as shoulder dystocia [31] or vacuum application [32] or for didactic purposes [33].

Another debated element is the degree of realism of these high-fidelity simulations which can interfere with the final result (i.e. the execution time). In our case, this objection is only partially true. To enhance the realistic effect, all the simulations were performed without any prior warning to the staff and during daily work. Most of the operators only became aware that it was a simulation during or at the end of the simulation. In our institute, simulations are usually performed during work. Therefore, although the participants could hypothesize that it could be a simulation, they were not aware of what kind of urgency it was until they arrived on site.

Based on our research, we can conclude that the implementation of simple and immediate operational protocols such as our “Five Actions for Five People” brings significant benefits both in terms of improving the quality of assistance and in the perception of staff who work as a team. Even if it is difficult to demonstrate an effect in the clinical practice due to the small number of cases, it is desirable that such procedures are widely introduced and confirmed by ad hoc simulation studies.

## Precis

“5 actions for 5 people” protocol improves the performance of the delivery room team and the quality of the operator's participation in urgency.

## Data Availability

The datasets used and/or analyzed during the current study are available from the corresponding author on reasonable request.

## References

[1] Habek D. Forensic expertise in obstetrics and gynecology – Forensic expert experience. Eur J Obstet Gynecol Reprod Biol. 2021;256. 10.1016/j.ejogrb.2020.10.046 33161209

[2] Wray J. Review of the National Sentinel Caesarean Section Audit Report. Pract Midwife. 2001;4. 12026696

[3] Neuhaus C, Lutnæs DE, Bergström J (2022). Emergence of power and complexity in obstetric teamwork. PLoS ONE.

[4] Detlefs SE, Goffman D, Buttle RA, Crump CM, Thornburg LL, Foley MR (2022). Correlation between medical management and teamwork in multidisciplinary high-fidelity obstetrics simulations. Am J Obstet Gynecol MFM.

[5] Leung TY, Lao TT. Timing of caesarean section according to urgency.Best Pract Res Clin Obstet Gynaecol. 2013;27. 10.1016/j.bpobgyn.2012.09.005 23116716

[6] RCOG. Good Practice No. 11 Classification of urgency of caesarean section – a continuum of risk. Good Practice No 11. 2010.

[7] Lucas DN, Yentis SM, Kinsella SM, Holdcroft A, May AE, Wee M et al. Urgency of caesarean section: A new classification. J R Soc Med. 2000;93. 10.1177/014107680009300703 PMC1298057 10928020

[8] Dupuis O, Sayegh I, Decullier E, Dupont C, Clément HJ, Berland M et al. Red, orange and green Caesarean sections: A new communication tool for on-call obstetricians. Eur J Obstet Gynecol Reprod Biol. 2008;140. 10.1016/j.ejogrb.2008.04.003 18495322

[9] Bloom SL, Leveno KJ, Gilbert S, Spong CY. Decision-to-incision times and maternal and infant outcomes [5]. Obstet Gynecol. 2006;108. 10.1097/01.AOG.0000224693.07785.14 16816049

[10] Schauberger CW. Decision-to-incision times and maternal and infant outcomes [4]. Obstet Gynecol. 2006;108. 10.1097/01.AOG.0000244982.46510.a4 17077260

[11] Dupuis O. Decision-to-incision times and maternal and infant outcomes [3]. Obstet Gynecol. 2006;108. 10.1097/01.AOG.0000244985.03231.21 17077258

[12] Rouse DJ. Decision-to-incision times and maternal and infant outcomes: Commentary. Obstet Gynecol Surv. 2006;61. 10.1097/01.AOG.0000224693.07785.14 16816049

[13] Bloom SL, Leveno KJ, Spong CY, Gilbert S, Hauth JC, Landon MB et al. Decision-to-incision times and maternal and infant outcomes. Obstet Gynecol. 2006;108. 10.1097/01.AOG.0000224693.07785.14 16816049

[14] van de Fransen AF, Banga FR, Mol BWJ, Oei SG (2020). Multi-professional simulation-based team training in obstetric emergencies for improving patient outcomes and trainees’ performance. Cochrane Database Syst Rev.

[15] Stanton NA, Salmon PM, Walker GH, Salas E, Hancock PA (2017). State-of-science: situation awareness in individuals, teams and systems. Ergonomics.

[16] Salas E, Wilson KA, Murphy CE, King H, Salisbury M (2008). Communicating, coordinating, and cooperating when lives depend on it: tips for teamwork. Jt Comm J Qual Patient Saf.

[17] Johnson SS, Grossman R, Miller JP, Christfort K, Traylor AM, Schweissing E (2021). Knowing well, being well: well-being born of understanding: the Science of Teamwork. Am J Health Promot.

[18] Leung TY, Lao TT (2013). Timing of caesarean section according to urgency. Best Pract Res Clin Obstet Gynaecol.

[19] of Pediatrics AA, of Obstetricians and Gynecologists AC, of Dimes Birth Defects. Foundation M, editors. Guidelines for perinatal care. 6th ed. Elk Grove Village, IL : Washington, DC: American Academy of Pediatrics ; American College of Obstetricians and Gynecologists; 2007.

[20] Schauberger CW, Chauhan SP. Emergency cesarean section and the 30-minute rule: Definitions. Am J Perinatol. 2009;26. 10.1055/s-0028-1103033 19031352

[21] Bousleiman S, Rouse DJ, Gyamfi-Bannerman C, Huang Y, D’alton ME, Siddiq Z et al. Decision to Incision and Risk for Fetal Acidemia, Low Apgar Scores, and Hypoxic Ischemic Encephalopathy. Am J Perinatol. 2022;39. 10.1055/s-0040-1717068 32957140

[22] Khan R, Hinshaw K. We are only human – Effective training in human factors. Best Pract Res Clin Obstet Gynaecol. 2022;80. 10.1016/j.bpobgyn.2022.02.003 35260355

[23] Kohn L, Coorigan J. To err is human: Building a safer health system. Summary. 1999. 25077248

[24] Kohn L, Corrigan J, Donaldson MS. Committee on Quality of Health Care in America. To err is human: building a Safer Health System. National Academy Press; 1999. 25077248

[25] Lacerenza CN, Marlow SL, Tannenbaum SI, Salas E (2018). Team development interventions: evidence-based approaches for improving teamwork. Am Psychol.

[26] Issenberg SB, McGaghie WC, Petrusa ER, Gordon DL, Scalese RJ. Features and uses of high-fidelity medical simulations that lead to effective learning: A BEME systematic review. Med Teach. 2005;27. 10.1080/01421590500046924 16147767

[27] Epstein RM, Hundert EM. Defining and assessing professional competence. JAMA. 2002;287. 10.1001/jama.287.2.226 11779266

[28] Cook DA, Hatala R, Brydges R, Zendejas B, Szostek JH, Wang AT et al. Technology-enhanced simulation for health professions education: A systematic review and meta-analysis. JAMA. 2011;306. 10.1001/jama.2011.1234 21900138

[29] Baker DP, Day R, Salas E. Teamwork as an essential component of high-reliability organizations. Health Serv Res. 2006;41 4 II. 10.1111/j.1475-6773.2006.00566.x PMC1955345 16898980

[30] Shapiro MJ, Morey JC, Small SD, Langford V, Kaylor CJ, Jagminas L et al. Simulation based teamwork training for emergency department staff: Does it improve clinical team performance when added to an existing didactic teamwork curriculum? Qual Saf Health Care. 2004;13. 10.1136/qshc.2003.005447 PMC1743923 15576702

[31] Mannella P, Palla G, Cuttano A, Boldrini A, Simoncini T. Effect of high-fidelity shoulder dystocia simulation on emergency obstetric skills and crew resource management skills among residents. Int J Gynecol Obstet. 2016;135. 10.1016/j.ijgo.2016.06.023 27622684

[32] Mannella P, Giordano M, Guevara MMM, Giannini A, Russo E, Pancetti F (2021). Simulation training program for vacuum application to improve technical skills in vacuum-assisted vaginal delivery. BMC Pregnancy Childbirth.

[33] Mannella P, Antonelli R, Montt-Guevara MM, Caretto M, Palla G, Giannini A et al. Simulation of childbirth improves clinical management capacity and self-confidence in medical students. BMJ Simul Technol Enhanc Learn. 2018;4. 10.1136/bmjstel-2017-000259 PMC8936910 35519004

